# 2-(2-Methyl-1,3-dioxolan-2-yl)-1,1-diphenyl­ethanol

**DOI:** 10.1107/S1600536809053227

**Published:** 2009-12-24

**Authors:** Dennis P. Arnold, John C. McMurtrie

**Affiliations:** a Chemistry, Queensland University of Technology, 2 George St, Brisbane, Queensland 4001, Australia

## Abstract

The mol­ecules of the title compound, C_18_H_20_O_3_, display an intra­molecular O—H⋯O hydrogen bond between the hydr­oxy donor and a ketal O-atom acceptor. In the crystal, inter­molecular C—H⋯π inter­actions connect adjacent mol­ecules into chains parallel to the *b* axis.

## Related literature

For the preparation of the title compound, see: Paulson *et al.* (1973 ▶). 
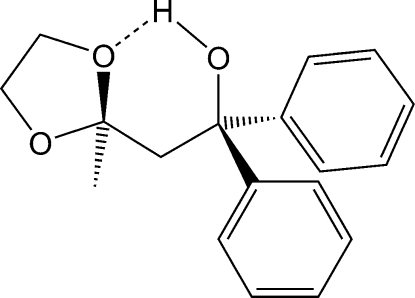


## Experimental

### 

#### Crystal data

C_18_H_20_O_3_
*M* *_r_* = 284.34
Monoclinic, 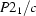

*a* = 5.7961 (4) Å
*b* = 8.8271 (7) Å
*c* = 29.754 (2) Å
β = 92.150 (7)°
*V* = 1521.26 (19) Å^3^
*Z* = 4
Mo *K*α radiation
μ = 0.08 mm^−1^
*T* = 173 K
0.68 × 0.35 × 0.09 mm

#### Data collection

Oxford Diffraction Gemini diffractometer
Absorption correction: multi-scan (*CrysAlis PRO*; Oxford Diffraction, 2007 ▶) *T*_min_ = 0.974, *T*_max_ = 1.000
5871 measured reflections
3407 independent reflections
2458 reflections with *I* > 2σ(*I*)
*R*_int_ = 0.017

#### Refinement

*R*[*F* ^2^ > 2σ(*F* ^2^)] = 0.041
*wR*(*F* ^2^) = 0.100
*S* = 1.03
3407 reflections
194 parameters
1 restraint
H atoms treated by a mixture of independent and constrained refinement
Δρ_max_ = 0.23 e Å^−3^
Δρ_min_ = −0.19 e Å^−3^

### 

Data collection: *CrysAlis CCD* (Oxford Diffraction, 2007 ▶); cell refinement: *CrysAlis RED* (Oxford Diffraction, 2007 ▶); data reduction: *CrysAlis RED*; program(s) used to solve structure: *SIR97* (Altomare *et al.*, 1999 ▶); program(s) used to refine structure: *SHELXL97* (Sheldrick, 2008 ▶); molecular graphics: *ORTEP-3* (Farrugia, 1997 ▶); software used to prepare material for publication: *publCIF* (Westrip, 2009 ▶).

## Supplementary Material

Crystal structure: contains datablocks I, global. DOI: 10.1107/S1600536809053227/jh2121sup1.cif

Structure factors: contains datablocks I. DOI: 10.1107/S1600536809053227/jh2121Isup2.hkl

Additional supplementary materials:  crystallographic information; 3D view; checkCIF report

## Figures and Tables

**Table 1:** Hydrogen-bond geometry (Å, °)

*D*—H⋯*A*	*D*—H	H⋯*A*	*D*⋯*A*	*D*—H⋯*A*
O3—H1*O*⋯O2	0.94 (1)	1.81 (1)	2.6820 (12)	153 (1)

**Fig. 1.**
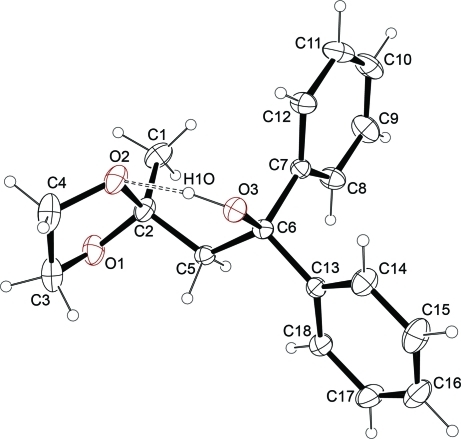
ORTEP depiction of the molecular structure with atom numbering scheme. Ellipsoids are drawn at the 40% probability level. The intramolecular hydrogen bond (O3—H···O2) is indicated by a dashed line.

**Fig. 2.**
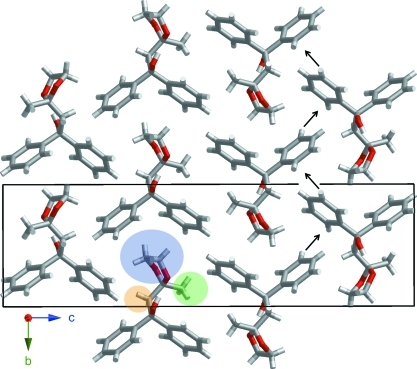
Crystal packing detail viewed parallel to the a axis. CH···π (edge to face) interactions propagate parallel to the b axis (black arrows). The arrangement of the aliphatic components (methyl green, ethylene orange and ketal blue) between four phenyl rings is indicated.

## supplementary crystallographic information

### Comment

The molecular stucture of the title compound, (I), is illustrated in Fig. 1. There is an intramolecular hydrogen bond between the hydroxy moiety and one of the ketal oxygen atoms (O3—H1O···O2, oxygen-oxygen distance 2.6820 (12) Å, O—H···O angle 153 (1)°). The presence of the hydrogen bond results in a loss of the average mirror symmetry and as a result the molecular conformer is chiral at C2. Both hands of the conformer are present in the structure as implied by the centrosymmetric space symmetry. The ^1^H NMR spectrum (room temperature) is indicative of the average conformation indicating that rearrangement in the solution state is rapid on the NMR timescale.

The molecules of (I) are arranged in chains that propagate parallel to the *b* axis *via* intermolecular CH···π interactions as illustrated in Fig. 2 (C15—H15_edge_···C13—C18_plane_ distance 2.96 Å). Interestingly, these are the only significant aryl-aryl interactions. The aliphatic components of the molecule including the methyl, methylene and ketal groups, completely occupy the space between the two phenyl rings (highlighted in Fig. 2) in which π interactions would be expected to occur. Adjacent chains are connected by weakly interacting aliphatic-CH···π interactions in addition to the omnipresent van der Waals forces.

### Experimental

The title compound was prepared by the procedure reported by Paulson *et al.* (1973). Large crystalline plates were obtained from methanol/water by vapour diffusion. NMR ^1^H (300 MHz, CDCl_3_) 7.53 (m, 4H, *ortho*-H), 7.30 (m, 4H, *meta*-H), 7.18 (tt, 2H, *para*-H), 5.39 (s, 1H, OH), 3.9–3.6 (symmetrical multiplets, AA'BB', 4H, ketal ring H), 2.84 (s, 2H, CH_2_), 1.07 (s, 3H, CH_3_).

### Refinement

C-bound H atoms were included in idealized positions and refined using a riding model approximation with methylene, methyl and aromatic bond lengths fixed at 0.99, 0.98 and 0.95 Å, respectively. *U*_iso_(H) values were fixed at 1.2*U*_eq_ of the parent C atoms for methylene and aromatic H atoms and 1.5*U*_eq_ of the parent C atoms for methyl H atoms. The hydroxy H atom was located in a Fourier difference map and refined with an O—H bond length restraint of 0.98 Å and with *U*_iso_ fixed at 1.5*U*_eq_ of the parent O atom.

### Crystal data

**Table:**

C_18_H_20_O_3_	*F*(000) = 608
*M**_r_* = 284.34	*D*_x_ = 1.241 Mg m^−^^3^
Monoclinic, *P*2_1_/*c*	Mo *K*α radiation, λ = 0.71073 Å
*a* = 5.7961 (4) Å	Cell parameters from 2617 reflections
*b* = 8.8271 (7) Å	θ = 3.4–28.6°
*c* = 29.754 (2) Å	µ = 0.08 mm^−^^1^
β = 92.150 (7)°	*T* = 173 K
*V* = 1521.26 (19) Å^3^	Plate, colourless
*Z* = 4	0.68 × 0.35 × 0.09 mm

### Data collection

**Table:**

Oxford Diffraction Gemini diffractometer	3407 independent reflections
Radiation source: Enhance (Mo) X-ray Source	2458 reflections with *I* > 2σ(*I*)
graphite	*R*_int_ = 0.017
Detector resolution: 16.0774 pixels mm^-1^	θ_max_ = 28.7°, θ_min_ = 3.5°
ω scans	*h* = −4→7
Absorption correction: multi-scan (*CrysAlis PRO*; Oxford Diffraction, 2007)	*k* = −11→10
*T*_min_ = 0.974, *T*_max_ = 1.000	*l* = −37→37
5871 measured reflections	

### Refinement

**Table:**

Refinement on *F*^2^	Primary atom site location: structure-invariant direct methods
Least-squares matrix: full	Secondary atom site location: difference Fourier map
*R*[*F*^2^ > 2σ(*F*^2^)] = 0.041	Hydrogen site location: inferred from neighbouring sites
*wR*(*F*^2^) = 0.100	H atoms treated by a mixture of independent and constrained refinement
*S* = 1.03	*w* = 1/[σ^2^(*F*_o_^2^) + (0.0523*P*)^2^] where *P* = (*F*_o_^2^ + 2*F*_c_^2^)/3
3407 reflections	(Δ/σ)_max_ = 0.001
194 parameters	Δρ_max_ = 0.23 e Å^−^^3^
1 restraint	Δρ_min_ = −0.19 e Å^−^^3^

### Special details

**Table:**

Experimental. CrysAlisPro, Oxford Diffraction Ltd., Version 1.171.33.52 (release 06-11-2009 CrysAlis171 .NET) (compiled Nov 6 2009,16:24:50) Empirical absorption correction using spherical harmonics, implemented in SCALE3 ABSPACK scaling algorithm.
Geometry. All e.s.d.'s (except the e.s.d. in the dihedral angle between two l.s. planes) are estimated using the full covariance matrix. The cell e.s.d.'s are taken into account individually in the estimation of e.s.d.'s in distances, angles and torsion angles; correlations between e.s.d.'s in cell parameters are only used when they are defined by crystal symmetry. An approximate (isotropic) treatment of cell e.s.d.'s is used for estimating e.s.d.'s involving l.s. planes.
Refinement. Refinement of *F*^2^ against ALL reflections. The weighted *R*-factor *wR* and goodness of fit *S* are based on *F*^2^, conventional *R*-factors *R* are based on *F*, with *F* set to zero for negative *F*^2^. The threshold expression of *F*^2^ > σ(*F*^2^) is used only for calculating *R*-factors(gt) *etc*. and is not relevant to the choice of reflections for refinement. *R*-factors based on *F*^2^ are statistically about twice as large as those based on *F*, and *R*- factors based on ALL data will be even larger.

### Fractional atomic coordinates and isotropic or equivalent isotropic displacement parameters (Å^2^)

**Table:**

	*x*	*y*	*z*	*U*_iso_*/*U*_eq_	
C1	0.9148 (2)	0.35245 (18)	0.06596 (5)	0.0381 (4)	
H1A	0.9150	0.2620	0.0468	0.057*	
H1B	1.0740	0.3862	0.0720	0.057*	
H1C	0.8270	0.4333	0.0506	0.057*	
C2	0.8042 (2)	0.31506 (15)	0.10991 (4)	0.0276 (3)	
C3	0.7536 (3)	0.10241 (17)	0.15222 (6)	0.0450 (4)	
H3A	0.7242	0.1454	0.1822	0.054*	
H3B	0.8023	−0.0046	0.1558	0.054*	
C4	0.5432 (3)	0.11605 (17)	0.12092 (6)	0.0465 (4)	
H4A	0.5403	0.0347	0.0980	0.056*	
H4B	0.3994	0.1112	0.1378	0.056*	
C5	0.8041 (2)	0.44391 (13)	0.14479 (4)	0.0214 (3)	
H5A	0.9611	0.4883	0.1463	0.026*	
H5B	0.7780	0.3976	0.1745	0.026*	
C6	0.63093 (18)	0.57549 (14)	0.13839 (4)	0.0193 (3)	
C7	0.6771 (2)	0.67148 (14)	0.09647 (4)	0.0213 (3)	
C8	0.8846 (2)	0.74953 (15)	0.09357 (4)	0.0300 (3)	
H8	0.9982	0.7406	0.1173	0.036*	
C9	0.9288 (2)	0.83969 (17)	0.05699 (5)	0.0387 (4)	
H9	1.0708	0.8931	0.0559	0.046*	
C10	0.7661 (3)	0.85235 (18)	0.02188 (5)	0.0402 (4)	
H10	0.7960	0.9139	−0.0034	0.048*	
C11	0.5610 (3)	0.77505 (18)	0.02399 (5)	0.0410 (4)	
H11	0.4495	0.7829	−0.0001	0.049*	
C12	0.5150 (2)	0.68539 (15)	0.06104 (4)	0.0306 (3)	
H12	0.3719	0.6333	0.0622	0.037*	
C13	0.6400 (2)	0.68055 (13)	0.17969 (4)	0.0208 (3)	
C14	0.4531 (2)	0.77607 (15)	0.18647 (5)	0.0318 (3)	
H14	0.3254	0.7752	0.1655	0.038*	
C15	0.4502 (3)	0.87249 (17)	0.22329 (5)	0.0413 (4)	
H15	0.3211	0.9368	0.2273	0.050*	
C16	0.6344 (3)	0.87537 (17)	0.25425 (5)	0.0391 (4)	
H16	0.6322	0.9406	0.2796	0.047*	
C17	0.8203 (3)	0.78268 (16)	0.24777 (5)	0.0388 (4)	
H17	0.9478	0.7846	0.2688	0.047*	
C18	0.8250 (2)	0.68578 (15)	0.21082 (4)	0.0289 (3)	
H18	0.9556	0.6228	0.2069	0.035*	
O1	0.92307 (16)	0.18920 (10)	0.12962 (3)	0.0369 (3)	
O2	0.56966 (15)	0.26253 (10)	0.10043 (3)	0.0344 (2)	
O3	0.39885 (13)	0.51655 (10)	0.13579 (3)	0.0244 (2)	
H1O	0.409 (2)	0.4207 (11)	0.1220 (4)	0.037*	

### Atomic displacement parameters (Å^2^)

**Table:**

	*U*^11^	*U*^22^	*U*^33^	*U*^12^	*U*^13^	*U*^23^
C1	0.0378 (8)	0.0446 (9)	0.0321 (8)	0.0005 (7)	0.0053 (6)	−0.0139 (7)
C2	0.0236 (6)	0.0255 (7)	0.0335 (7)	0.0007 (6)	−0.0033 (5)	−0.0071 (6)
C3	0.0514 (10)	0.0226 (8)	0.0614 (10)	−0.0015 (7)	0.0062 (8)	−0.0012 (7)
C4	0.0409 (9)	0.0264 (8)	0.0726 (12)	−0.0062 (7)	0.0069 (8)	−0.0054 (8)
C5	0.0208 (6)	0.0232 (7)	0.0199 (6)	−0.0019 (5)	−0.0009 (5)	−0.0005 (5)
C6	0.0166 (6)	0.0227 (6)	0.0188 (6)	−0.0030 (5)	0.0002 (5)	0.0006 (5)
C7	0.0229 (6)	0.0222 (6)	0.0190 (6)	0.0022 (5)	0.0023 (5)	−0.0001 (5)
C8	0.0259 (7)	0.0365 (8)	0.0275 (7)	−0.0024 (6)	0.0009 (5)	0.0066 (6)
C9	0.0338 (8)	0.0415 (9)	0.0414 (8)	−0.0036 (7)	0.0099 (6)	0.0131 (7)
C10	0.0485 (9)	0.0439 (9)	0.0291 (7)	0.0100 (7)	0.0122 (7)	0.0169 (7)
C11	0.0451 (9)	0.0525 (10)	0.0248 (7)	0.0059 (8)	−0.0052 (6)	0.0090 (7)
C12	0.0299 (7)	0.0352 (8)	0.0265 (7)	0.0000 (6)	−0.0039 (5)	0.0030 (6)
C13	0.0235 (6)	0.0196 (6)	0.0194 (6)	−0.0038 (5)	0.0036 (5)	0.0014 (5)
C14	0.0269 (7)	0.0347 (8)	0.0340 (7)	0.0012 (6)	0.0028 (6)	−0.0068 (6)
C15	0.0371 (8)	0.0392 (8)	0.0487 (9)	0.0027 (7)	0.0148 (7)	−0.0157 (8)
C16	0.0486 (9)	0.0384 (8)	0.0311 (8)	−0.0096 (7)	0.0112 (7)	−0.0133 (7)
C17	0.0465 (9)	0.0411 (9)	0.0281 (7)	−0.0070 (7)	−0.0071 (6)	−0.0071 (7)
C18	0.0312 (7)	0.0279 (7)	0.0273 (7)	0.0007 (6)	−0.0027 (5)	−0.0023 (6)
O1	0.0331 (5)	0.0252 (5)	0.0521 (6)	0.0051 (4)	−0.0017 (5)	−0.0042 (5)
O2	0.0286 (5)	0.0299 (5)	0.0443 (6)	−0.0050 (4)	−0.0044 (4)	−0.0100 (5)
O3	0.0187 (4)	0.0257 (5)	0.0288 (5)	−0.0043 (4)	0.0015 (3)	−0.0015 (4)

### Geometric parameters (Å, °)

**Table:**

C1—C2	1.5145 (18)	C8—C9	1.3802 (18)
C1—H1A	0.9800	C8—H8	0.9500
C1—H1B	0.9800	C9—C10	1.385 (2)
C1—H1C	0.9800	C9—H9	0.9500
C2—O1	1.4222 (16)	C10—C11	1.374 (2)
C2—O2	1.4539 (15)	C10—H10	0.9500
C2—C5	1.5396 (17)	C11—C12	1.3912 (19)
C3—O1	1.4330 (17)	C11—H11	0.9500
C3—C4	1.511 (2)	C12—H12	0.9500
C3—H3A	0.9900	C13—C18	1.3914 (18)
C3—H3B	0.9900	C13—C14	1.3932 (17)
C4—O2	1.4402 (17)	C14—C15	1.3879 (19)
C4—H4A	0.9900	C14—H14	0.9500
C4—H4B	0.9900	C15—C16	1.384 (2)
C5—C6	1.5422 (17)	C15—H15	0.9500
C5—H5A	0.9900	C16—C17	1.372 (2)
C5—H5B	0.9900	C16—H16	0.9500
C6—O3	1.4416 (13)	C17—C18	1.3940 (18)
C6—C13	1.5387 (16)	C17—H17	0.9500
C6—C7	1.5398 (16)	C18—H18	0.9500
C7—C12	1.3908 (18)	O3—H1O	0.943 (8)
C7—C8	1.3914 (17)		
			
C2—C1—H1A	109.5	C8—C7—C6	119.97 (11)
C2—C1—H1B	109.5	C9—C8—C7	121.39 (13)
H1A—C1—H1B	109.5	C9—C8—H8	119.3
C2—C1—H1C	109.5	C7—C8—H8	119.3
H1A—C1—H1C	109.5	C8—C9—C10	119.99 (13)
H1B—C1—H1C	109.5	C8—C9—H9	120.0
O1—C2—O2	105.42 (10)	C10—C9—H9	120.0
O1—C2—C1	108.18 (10)	C11—C10—C9	119.42 (13)
O2—C2—C1	108.96 (11)	C11—C10—H10	120.3
O1—C2—C5	108.16 (10)	C9—C10—H10	120.3
O2—C2—C5	110.03 (10)	C10—C11—C12	120.71 (13)
C1—C2—C5	115.59 (11)	C10—C11—H11	119.6
O1—C3—C4	102.69 (13)	C12—C11—H11	119.6
O1—C3—H3A	111.2	C7—C12—C11	120.43 (13)
C4—C3—H3A	111.2	C7—C12—H12	119.8
O1—C3—H3B	111.2	C11—C12—H12	119.8
C4—C3—H3B	111.2	C18—C13—C14	117.86 (12)
H3A—C3—H3B	109.1	C18—C13—C6	123.64 (11)
O2—C4—C3	103.66 (11)	C14—C13—C6	118.50 (11)
O2—C4—H4A	111.0	C15—C14—C13	121.21 (13)
C3—C4—H4A	111.0	C15—C14—H14	119.4
O2—C4—H4B	111.0	C13—C14—H14	119.4
C3—C4—H4B	111.0	C16—C15—C14	120.30 (13)
H4A—C4—H4B	109.0	C16—C15—H15	119.8
C2—C5—C6	119.34 (10)	C14—C15—H15	119.8
C2—C5—H5A	107.5	C17—C16—C15	119.07 (13)
C6—C5—H5A	107.5	C17—C16—H16	120.5
C2—C5—H5B	107.5	C15—C16—H16	120.5
C6—C5—H5B	107.5	C16—C17—C18	121.00 (13)
H5A—C5—H5B	107.0	C16—C17—H17	119.5
O3—C6—C13	105.32 (8)	C18—C17—H17	119.5
O3—C6—C7	110.19 (9)	C13—C18—C17	120.55 (12)
C13—C6—C7	108.27 (10)	C13—C18—H18	119.7
O3—C6—C5	109.66 (10)	C17—C18—H18	119.7
C13—C6—C5	110.67 (9)	C2—O1—C3	106.33 (10)
C7—C6—C5	112.48 (9)	C4—O2—C2	108.53 (10)
C12—C7—C8	118.05 (11)	C6—O3—H1O	105.7 (8)
C12—C7—C6	121.97 (11)		
			
O1—C3—C4—O2	−30.56 (14)	C7—C6—C13—C18	−104.24 (13)
O1—C2—C5—C6	−162.39 (10)	C5—C6—C13—C18	19.46 (15)
O2—C2—C5—C6	−47.72 (14)	O3—C6—C13—C14	−42.13 (14)
C1—C2—C5—C6	76.20 (14)	C7—C6—C13—C14	75.73 (13)
C2—C5—C6—O3	57.28 (13)	C5—C6—C13—C14	−160.56 (10)
C2—C5—C6—C13	173.04 (10)	C18—C13—C14—C15	−0.58 (19)
C2—C5—C6—C7	−65.72 (14)	C6—C13—C14—C15	179.44 (12)
O3—C6—C7—C12	−4.07 (16)	C13—C14—C15—C16	−0.1 (2)
C13—C6—C7—C12	−118.77 (13)	C14—C15—C16—C17	0.6 (2)
C5—C6—C7—C12	118.62 (12)	C15—C16—C17—C18	−0.4 (2)
O3—C6—C7—C8	174.87 (10)	C14—C13—C18—C17	0.75 (19)
C13—C6—C7—C8	60.17 (14)	C6—C13—C18—C17	−179.28 (11)
C5—C6—C7—C8	−62.43 (15)	C16—C17—C18—C13	−0.3 (2)
C12—C7—C8—C9	0.7 (2)	O2—C2—O1—C3	−29.34 (13)
C6—C7—C8—C9	−178.28 (12)	C1—C2—O1—C3	−145.78 (11)
C7—C8—C9—C10	−0.8 (2)	C5—C2—O1—C3	88.33 (12)
C8—C9—C10—C11	0.3 (2)	C4—C3—O1—C2	37.24 (14)
C9—C10—C11—C12	0.4 (2)	C3—C4—O2—C2	13.39 (14)
C8—C7—C12—C11	0.0 (2)	O1—C2—O2—C4	9.01 (13)
C6—C7—C12—C11	178.97 (12)	C1—C2—O2—C4	124.92 (12)
C10—C11—C12—C7	−0.6 (2)	C5—C2—O2—C4	−107.39 (12)
O3—C6—C13—C18	137.90 (11)		

### Hydrogen-bond geometry (Å, °)

**Table:**

*D*—H···*A*	*D*—H	H···*A*	*D*···*A*	*D*—H···*A*
O3—H1O···O2	0.94 (1)	1.81 (1)	2.6820 (12)	153 (1)
